# Relationship of spontaneous microembolic signals to risk stratification, recurrence, severity, and mortality of ischemic stroke: a prospective study

**DOI:** 10.1186/s13089-020-0156-1

**Published:** 2020-02-11

**Authors:** Rodrigo Bazan, Gustavo José Luvizutto, Gabriel Pereira Braga, Silméia Garcia Zanati Bazan, João Carlos Hueb, Carlos Clayton Macedo de Freitas, Pedro Tadao Hamamoto Filho, Gabriel Pinheiro Módolo, André Petean Trindade, Marcone Lima Sobreira, Hélio Rubens de Carvalho Nunes, João Pereira Leite, Octávio Marques Pontes-Neto

**Affiliations:** 1 Department of Neurology, Psychology and Psychiatry, Botucatu Medical School, UNESP–Univ Estadual Paulista, Campus de Botucatu, Distrito de Rubião Jr, s/n, Botucatu, Brazil; 2 Department of Applied Physical Therapy, Institute of Health Sciences, UFTM–Univ Federal do Triângulo Mineiro, Uberaba, Brazil; 3 Department of Internal Medicine, Botucatu Medical School, UNESP–Univ Estadual Paulista, Botucatu, Brazil; 4 Department of Tropical Diseases and Imaging Diagnosis, Botucatu Medical School, UNESP–Univ Estadual Paulista, Botucatu, Brazil; 5 Department of Surgery and Orthopedics, Botucatu Medical School, UNESP–Univ Estadual Paulista, Botucatu, Brazil; 6 Department of Neuroscience and Behavior, Ribeirão Preto School of Medicine, USP–Univ São Paulo, Ribeirão Preto, Brazil

**Keywords:** Brain embolism, Prognosis, Risk factors, Stroke, Transcranial Doppler ultrasonography

## Abstract

**Introduction:**

The presence of microembolic signals (MES) during the acute phase of stroke is poorly understood, and its role and clinical application in relation to risk stratification and prognosis in patients remain uncertain. We assessed the prevalence of spontaneous MES in acute stroke and their relationship with risk stratification, stroke recurrence, morbidity, and mortality.

**Patients and methods:**

This was a prospective cohort study conducted in the Stroke Unit. The MES presence was evaluated by transcranial Doppler (TCD) in patients with ischemic stroke within 48 h. The outcomes (risk stratification, morbidity, mortality, and recurrence of a stroke) were followed up for 6 months. The relationship between risk stratification and MES was obtained by odds ratios and that between MES and stroke recurrence, morbidity, and mortality using multiple logistic regression; considering statistical significance at *P* < 0.05.

**Results:**

Of the 111 patients studied, 70 were men (63.1%) and 90 were white (81.1%), with a median age of 68 years. The MES frequency was 7%. There was a significant relationship between MES and symptomatic carotid disease (OR = 22.7; 95% CI 4.1–125.7; *P* < 0.001), a shorter time to monitoring (OR = 12.4; 95% CI  1.4–105.4; *P* = 0.02), and stroke recurrence (OR = 16.83; 95% CI 2.01–141; *P* = .009).

**Discussion:**

It was observed that the stroke recurrence adjusted for prior stroke was higher and earlier among patients with MES detection. In conclusion, MES demonstrated a significant correlation with symptomatic carotid disease and a shorter DELAY until monitoring, and could be a predictor for the early recurrence of stroke in the long term.

## Introduction

Transcranial Doppler (TCD) is an important tool used in health care to assess blood flow velocity in brain arteries, with extensive application in neurovascular clinical practice [1, 2]. TCD has potential diagnostic applications in locating embolic sources and long-term risk prediction of ischemic stroke [3]. The detection of embolic signals has been observed in a variety of potential embolic sources during patient assessment with TCD. Experimental studies have shown that this technique has a high sensitivity and specificity for the detection of various types of embolic elements [4, 5].

Data from many studies have demonstrated that the detection of spontaneous microembolic signals (MES) can predict the risk of stroke in patients with cardiac emboli or large artery disease, especially in cases of symptomatic internal carotid stenosis [6–10]. TCD is the only method that permits direct identification of embolic phenomena through the detection of an MES [11, 12].

The presence of MES on TCD during the acute phase of stroke is poorly understood, and its role and clinical application in relation to risk stratification and prognosis in patients remain uncertain, with no study having been conducted in the Latin American population. We assessed the hypothesis that investigating MES prevalence in the acute phase of stroke is related to risk stratification, stroke recurrence, disability, and death.

## Materials and methods

### Ethics approval and consent to participate

This trial was approved by a Committee for Ethics in Research involving human subjects from Ribeirão Preto School of Medicine. Upon inclusion, all patients or legal surrogates provided written informed consent in accordance with the Declaration of Helsinki II.

### Study design, setting, and participants

This was a prospective cohort study of consecutive adult patients of both sexes diagnosed with ischemic stroke of the anterior circulation during the first 48 h of ictus. This study was conducted in the Stroke Unit at Botucatu Medical School (UNESP) from January 2015 to March 2016. The inclusion criteria for monitoring included a diagnosis of ischemic stroke of the anterior circulation territory confirmed by neuroimaging examinations (CT or MRI) and by OSCP (The Oxford Community Stroke Project classification) within the first 48 h of ictus, age above 18 years, monitoring with TCD for a minimum duration of 30 min, and performance of phenotypic classification and stroke risk stratification by Trial of Org 10172 in Acute Stroke Treatment (TOAST), preferably in the first 3 months after inclusion in the study [13].

The study excluded patients with a diagnosis of hemorrhagic stroke or with the presence of other types of brain lesions of non-vascular etiology, those who did not have a temporal acoustic window (TAW) to undergo TCD, those who underwent any surgical procedure that impeded the realization of monitoring, or those lost during the 6-month follow-up. Also excluded were examinations of poor technical quality during TCD due to various artifacts during recording.

### Variables

#### Definition of risk factors

The risk factors evaluated were as follows: hypertension, smoking, obesity, diabetes mellitus (DM), at-risk drinkers, dyslipidemia, Chagas’ disease, prior stroke, congestive heart failure (CHF), prior acute myocardial infarction (AMI), coronary artery disease (CAD), depression, atrial fibrillation, atrial flutter, patent foramen ovale (PFO, confirmed with transthoracic and/or transesophageal echocardiogram), and extracardiac shunts. These data were collected from the clinical history of patients, such as previous use of antihypertensive medications, oral hypoglycemic agents or parenteral insulin, oral lipid-lowering drugs, or were confirmed clinically and by laboratory tests during hospitalization. Levels considered for confirmation were hypertension with systolic blood pressure ≥ 140/90 mmHg, dyslipidemia with cholesterol levels ≥ 240 mg/dL, diabetes mellitus with glycated hemoglobin level > 7%, obese patients with a body mass index ≥ 30 kg/m^2^, and a score > 8 in the Hospital Anxiety and Depression Scale (HADS) [14–17].

### TCD examination

A portable TCD device (DWL, Doppler Box model, Compumedics, Singen, Baden-Württemberg, Germany) was placed between the lateral margin of the orbit and the ear, above the zygomatic arch. Low-power pulsed-wave 2-MHz transducers, 1.7 cm in diameter, were used with TCD-8 software (Version 8.00 K) at a pulse repetition frequency of 6500 Hz and a programmable high-pass filter of 50–600 Hz. The transducers were fixed bilaterally to a helmet to monitor both middle cerebral arteries at 45–60 mm.

All patients were initially evaluated by two independent neurologists for the presence of a temporal acoustic window (TAW) by TCD. Patients with bilateral TAWs underwent examination with TCD for at least 30 min. The researchers were single blinded during monitoring to the risk stratification of the patients. By the time of TCD, intravenous solution dripping was interrupted.

### Microembolic signals detection

**Fig. 1:**
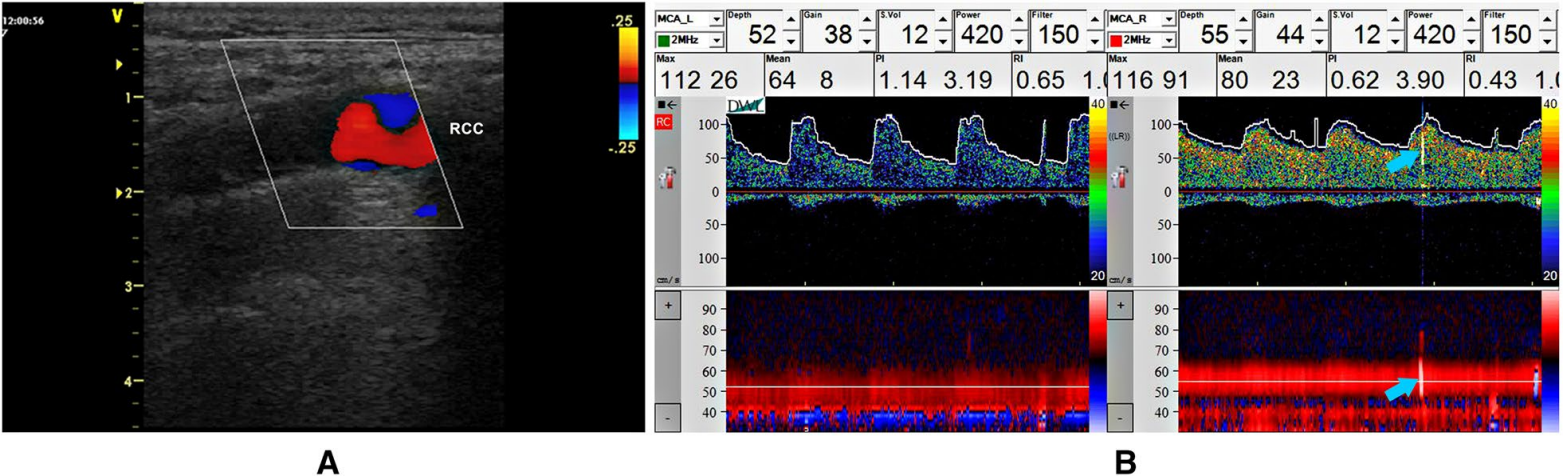
**a** Suggestive image of a plaque in the right common carotid artery (rcca); **b** in the same patient, spontaneous microembolic signal was detected by transcranial Doppler monitoring in acute phase of stroke. Blue arrows indicate MES in wave spectrum and in Mode M

To evaluate MES, the following characteristics were considered according to the microembolus detection consensus [12]: randomly occurring during the cardiac cycle, short (< 0.1 s), high intensity (3–9 dB) above the wave spectrum, and characteristic audible sound. We used the Power Doppler M in all tests to identify the arteries and aid in the identification of microembolic signals (Fig. 1). In general, monitoring took 45 min. Automatic detection of microembolic signals was used in association with visual and sound discretion of microemboli. All tests were recorded for at least 30 min, filed, and independently reviewed by two blinded investigators to identify MES [12]. The presence or absence of MES was reported, as well the number of MES for each patient.

### Clinical variables and risk stratification

The patients were classified according to TOAST, examination of brain imaging by computed tomography (CT) or magnetic resonance imaging (MRI), extensive laboratory examinations, electrocardiogram (ECG), transthoracic or transesophageal echocardiography, and duplex or CT angiography of the carotid and vertebrobasilar system. All patients underwent hospitalization in the Stroke Unit and received standard care according to the international protocols [18–20]. The degree of carotid stenosis was classified by the NASCET criteria, defined as < 50%, 50–69%, ≥ 70%, or occlusion [21]. TCD examination was performed in all patients to evaluate possible intracranial stenosis. MR angiography, CT angiography, and digital arteriography were performed on the suspicion of stenosis of intracranial or extracranial vessels. The examination of digital 24-h Holter monitoring was conducted in subjects over 55 years and suspected of paroxysmal atrial fibrillation.

### Follow-up

Outpatient follow-up time was 6 months after the onset of stroke and was performed by a blinded investigator. Disability was monitored using the modified Rankin Scale (mRS), with mRS 0–2 being classified as favorable and mRS 6 as death, and the recurrence of stroke was monitored throughout the study. Recurrence of stroke was documented by means of clinical and radiological evaluation.

### Primary outcome measures

Disability and death in 6 months.

### Secondary outcome measures

Recurrence of stroke in 6 months.

### Study size and sampling

We needed a minimum of 100 subjects to obtain a maximum sampling error of 7.5% and a confidence level of 95%. Type I and II error probabilities equaled 0.05 and 0.20, respectively, and it was estimated that the power to test the association between the presence of MES with stroke < 48 h post-ictus, atheromatous carotid, and symptomatic carotid artery disease was above 80%. For clinical follow-up data, it was estimated that the power to test the association between the presence of MES and recurrence, disability, and death was below 65%.

### Statistical methods

The interobserver agreement of the presence of MES was classified by Cohen’s *κ* coefficient. The risk stratification of patients with acute ischemic stroke and the relationship with MES had point and interval estimations of the odds ratios, depending on the statistically related independent variables calculated by Fisher’s exact test, taking into account confounding factors (thrombolysis, level of anticoagulation, and antiplatelet therapies) at monitoring. The selected variables with *P* < 0.05 in the univariate analysis were included in the multiple logistic regression to determine the independent variables associated with the presence of MES.

The relationship between morbidity and mortality in patients and MES (presence or absence and number for patient) during follow-up was evaluated using univariate analysis for potential confounders, such as age, sex, race, hypertension, NIHSS at admission, previous mRS, hemorrhagic transformation, smoking, obesity, diabetes, dyslipidemia, Chagas’ disease, prior stroke, prior AMI, CAD, depression, wake-up stroke, carotid dissection, carotid atheroma, symptomatic carotid disease, intracranial stenosis, atrial flutter, atrial fibrillation, cardiac heart failure (CHF), patent foramen ovale (PFO), extracardiac shunt, and thrombolysis. A subsequent multiple logistic regression analysis was performed with backward selection correcting for the effect of potential confounders.

The variables more strongly associated (*P* < 0.20) with the incidence rate of stroke recurrence were included in a Cox proportional regression model. Statistical analysis was carried out with SPSS software (Version 21.0, SPSS, Chicago, IL, USA).

## Results

**Fig. 2:**
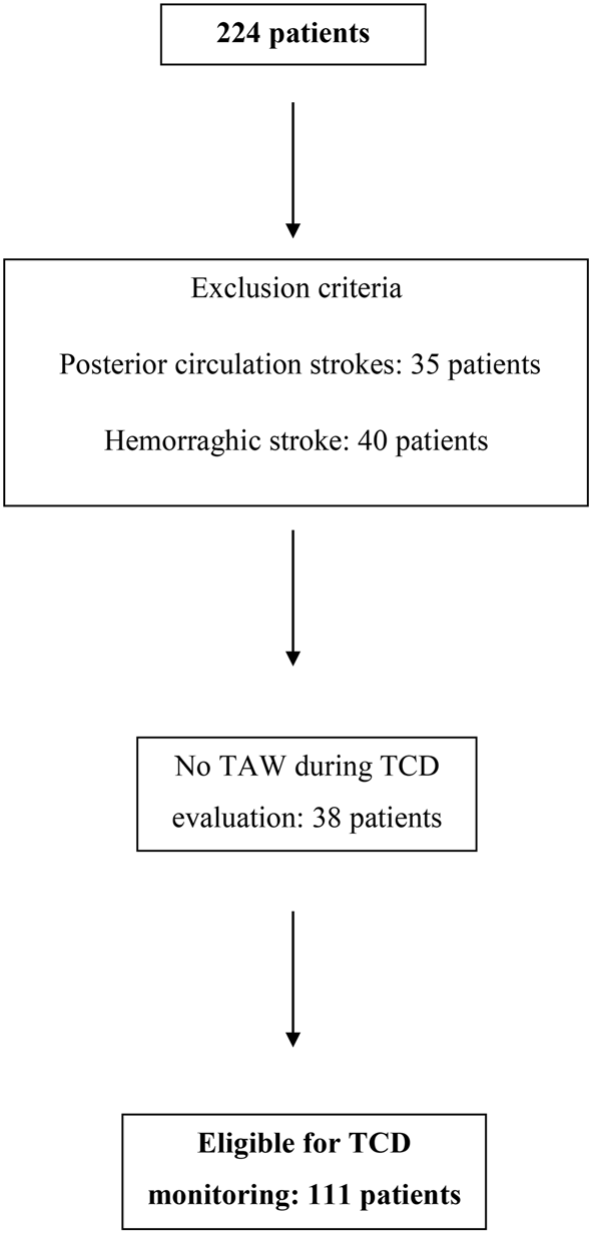
Flow chart

A total of 254 patients were screened in this study, with 105 excluded by the exclusion criteria. Only 149 patients were considered for monitoring, and 38 patients (26%) showed no TAW during TCD evaluation (Fig. 2).

A high level of concordance was demonstrated between the two experts who rated the presence of MES (*κ* = 0.95; *P* < 0.01, % agreement = 98%). The total number of patients monitored with TCD was 111; 70 were men (63.1%) and 90 were white (81.1%), with a median age of 68 years. Hypertension was found to be the main risk factor (83.8%). MES were found in eight patients (7%). Participant characteristics are displayed in Table 1.

**Table 1:** Demographic profile, key risk factors, and clinical and etiological classification of monitored patients

Variables	*n*	%
Demographic profile		
Male	70	63.1
Age (years)	68 (33–97)	
Race		
White	90	81.1
Black/Asian	21	19.9
Risk factors		
Hypertension	93	83.8
Smoking	60	54.1
Obesity	6	5.4
Diabetes	30	27
Dyslipidemia	17	15.3
Chagas disease	8	7.2
Prior stroke	36	32.4
Congestive heart failure	8	7.2
Prior AMI	11	9.9
CAD	12	10.8
Depression	6	5.4
Atrial fibrillation	29	26.1
Atrial flutter	4	3.6
PFO	2	1.8
Extracardiac shunt	1	0.9
Thrombolysis	26	23.4
Antiplatelets	67	60.0
Anticoagulation	3	2.7
Wake-up stroke	18	16.2
NHISS at admission	8 (0–28)	
OSCP		
TACS	25	22.5
PACS	56	50.4
LACS	30	27.0
TOAST		
Small-vessel occlusion	23	21.0
Large-artery atherosclerosis	11	9.9
Cardioembolism	31	28.0
Other determined etiology	5	4.5
Undetermined etiology	41	37

*AMI* acute myocardial infarction,* CAD* coronary artery disease,* NIHSS* National Institutes of Health Stroke Scale,* OSCP* The Oxford Community Stroke Project classification,* TACS* total anterior circulation syndrome,* PACS* partial anterior circulation syndrome,* LACS* lacunar syndrome

In relation to MES detection, thrombolytic therapy and the level of use of therapeutic oral anticoagulation (INR > 2) and antiplatelet therapies at the time of monitoring were analyzed as potential confounders. There was no statistically significant relationship to these variables. Demographic variables, such as sex and race, were also not statistically significant for MES detection.

MES detection was higher in patients with atherosclerotic carotid (OR = 15.5, 95% CI   3.2 to 74.7; *P* = 0.001) and symptomatic carotid artery disease (OR = 22.7; 95% CI  4.1 to 125.7; *P* = 0.001). MES were not detected in patients with small vessel disease or atrial fibrillation. MES detection was higher among patients who were monitored earlier in relation to the onset of symptoms of stroke (OR = 2.4, 95% CI 1.4 to 105.4; *P* = 0.02) (Table 2).

**Table 2:** Relationship between risk factors, etiology, and clinical presentation with MES presence

	MES	*P*
	*n* (%)	
Hypertension		
No (*n* = 18)	3 (17%)	0.119
Yes (*n* = 93)	5 (5%)	
Smoking		
No (*n* = 51)	4 (8%)	1.000
Yes (*n* = 60)	4 (7%)	
Obesity		
No (*n* = 105)	7 (7%)	0.369
Yes (*n* = 6)	1 (17%)	
Diabetes		
No (*n* = 81)	6 (7%)	1.000
Yes (*n* = 30)	2 (7%)	
Dyslipidemia		
No (*n* = 94)	6 (6%)	0.354
Yes (*n* = 17)	2 (12%)	
Chagas disease		
No (*n* = 103)	7 (7%)	0.462
Yes (*n* = 8)	1 (12.5%)	
Prior stroke		
No (*n* = 75)	7 (9%)	0.271
Yes (*n* = 36)	1 (3%)	
Cardiac heart failure		
No (n = 103)	8 (8%)	1.000
Yes (*n* = 8)	0 (0%)	
Prior AMI		
No (*n* = 100)	8 (8%)	1.000
Yes (*n* = 11)	0 (0%)	
CAD		
No (*n* = 99)	8 (8%)	0.595
Yes (*n* = 12)	0 (0%)	
Depression		
No (*n* = 105)	7 (6%)	0.369
Yes (*n* = 6)	1 (17%)	
TOAST		
Small-vessel occlusion (*n* = 23)	0 (0%)	<0.001
Large-artery atherosclerosis (*n* = 11)	5 (46%)	
Cardioembolism (*n* = 31)	0 (0%)	
Other determined etiology (*n* = 5)	2 (40%)	
Undetermined etiology (*n* = 41)	1 (2%)	
Ictus time < 24 h		
No (*n* = 67)	1 (1.5%)	0.006
Yes (*n* = 44)	7 (16%)	
Wake-up stroke		
No (*n* = 93)	6 (7%)	0.614
Yes (*n* = 18)	2 (11%)	
Carotid dissection		
No (*n* = 109)	7 (6%)	0.140
Yes (n = 2)	1 (50%)	
Carotid atheromatous		
No (*n* = 96)	3 (3%)	0.001
Yes (*n* = 15)	5 (33%)	
Symptomatic carotid		
No (*n* = 93)	2 (2%)	<0.001
Yes (*n* = 18)	6 (3%)	
Intracranial stenosis		
No (*n* = 105)	8 (7%)	1.000
Yes (*n* = 6)	0 (0%)	
Atrial flutter		
No (*n* = 107)	8 (8%)	1.000
Yes (*n* = 4)	0 (0%)	
Atrial fibrillation		
No (*n* = 82)	8 (10%)	0.108
Yes (*n* = 29)	0 (0%)	
PFO		
No (*n = *109)	8 (7%)	1.000
Yes (*n = *2)	0 (0%)	
Extracardiac shunt		
No (*n* = 110)	8 (7%)	1.000
Yes (*n* = 1)	1 (100%)	
Plaques in the aortic arch		
No (*n* = 109)	8 (7%)	1.000
Yes (*n* = 2)	0 (0%)	

Fisher exact test

Table 3 shows the association between MES and outcomes. The chance for favorable outcomes were reduced with increasing age (OR = 0.96; 95% CI 0.91 to 1.00, *P* = 0.036), increasing NIHSS at admission (OR = 0.80; 95% CI 0.73 to 0.87, *P* < 0.001), and higher prior mRS (OR = 0.31; 95% CI 0.14 to 0.66, *P* = 0.002). The probability for favorable outcomes tended to be higher in patients who smoked (OR = 2.83; 95% CI 0.98 to 8.15, *P* = 0.054). There was no significant relationship between MES and favorable outcomes (mRS 0–2) (OR = 2.4, 95% CI 0.36 to 16.36, *P* = 0.366). The probability of death was higher with increasing age (OR = 1.07; 95% CI 1.01 to 1.12, *P* = 0.014) and increasing NIHSS (OR = 1.16; 95% CI 1.07 to 1.26, *P* < 0.001). There was no significant relationship between MES and the occurrence of death (mRS 6) (OR = 2.27, 95% CI 0.19 to 26.50, *P* = 0.513).

**Table 3:** Simple and multiple regression logistics between confounder factors with outcomes

Variables	Simple regression (univariate analysis)						Multiple regression (multivariate analysis)					
	mRS 0–2		Death		Recurrence		mRS 0–2		Death		Recurrence	
	*n* (%)	*P*	*n* (%)	*P*	*n* (%)	*P*	*P*	OR (CI95%)	*P*	OR (IC95%)	*P*	OR (CI95%)
Sex		0.548		0.851		1.000						
Male	44 (62.9)		11 (15.7)		3 (2.4)							
Female	23 (56.1)		7 (17.1)		1 (4.3)							
Age (years)	–	0.002	–	0.005	–	0.986	0.036	0.96 (0.92–1.0)	0.014	1.07 (1.01–1.12)		
Race		0.366		0.187		1.000						
Caucasian	52 (57.8)		17 (18.9)		4 (4.4)							
Non-Caucasian	15 (75.7)		1 (5.0)		0							
Hypertension		0.132		0.46		1.000						
Yes (*n = *93)	59 (63.4)		13 (14.0)		4 (4.3)							
No (*n = *18)	8 (44.4)		5 (27.8)		0							
NIHSS at admission	–	< 0.001	–	<0.001	–	0.295	0.000	0.80 (0.73–0.87)	0.000	1.16 (1.07–1.26)		
Prior mRS	–	0.014	–	0.876	NC	–	0.002	0.31 (0.14–0.66)				
Hemorrhagic transformation		0.111		0.317		1.000						
Yes	2 (28.6)		2 (28.6)		0							
No	65 (62.5)		16 (15.4)		4 (3.8)							
Smoking		0.002		0.158		0.623	0.054	2.83 (0.98–8.15)				
Yes (*n = *60)	44 (73.3)		7 (11.7)		3 (5.0)							
No	23 (45.1)		11 (21.6)		1 (2.0)							
Obesity		0.680		1.000		1.000						
Yes (*n = *6)	3 (50.0)		1 (16.7)		0							
No (*n = *105)	64 (61.0)		17 (16.2)		4 (3.8)							
Diabetes		0.206		0.280		0.295						
Yes (*n = *30)	21 (70.0)		3 (18.5)		2 (6.7)							
No (*n = *81)	46 (56.8)		15 (10.0)		2 (2.5)							
Dyslipidemia		0.182		0.298		0.110						
Yes (*n = *17)	13 (76.5)		1 (5.9)		2 (11.8)							
No (*n = *94)	54 (57.4)		17 (18.1)		2 (2.1)							
Chagas disease		0.710		0.350		1.000						
Yes (*n = *8)	4 (50.0)		0		0							
No (*n = *103)	63 (61.2)		18 (17.5)		4 (3.9)							
Prior stroke		0.122		0.523		0.099						
Yes (*n = *36)	18 (50.0)		7 (19.4)		3 (8.3)							
No (*n = *75)	49 (65.3)		11 (14.7)		1 (1.3)							
Prior AMI		0.287		1.000		0.345						
Yes (*n = *11)	5 (45.5)		2 (18.2)		1 (9.1)							
No (*n = *100)	62 (62.0)		16 (16.0)		3 (3.0)							
CAD		0.879		0.687		0.057						
Yes (*n = *12)	7 (58.3)		1 (8.3)		2 (16.7)							
No (*n = *99)	60 (60.6)		17 (17.2)		2 (2.0)							
Depression		1.000		0.587		0.202						
Yes (*n = *6)	4 (60.6)		0		1 (16.7)							
No (*n = *105)	63 (50.0)		18 (17.1)		3 (2.9)							
Wake-up stroke		0.943		1.000		0.512						
Yes (*n = *18)	11 (61.1)		3 (16.7)		1 (5.6)							
No (*n = *93)	56 (60.2)		15 (16.1)		3 (3.2)							
Carotid dissection		1.000		1.000		1.000						
Yes (*n = *2)	1 (60.6)		0		0							
No (*n = *109)	66 (50.0)		18 (16.5)		4 (3.7)							
Carotid atheromatous		0.550		0.708		0.445						
Yes (*n = *15)	8 (53.3)		3 (20.0)		1 (6.7)							
No (*n = *96)	59 (61.5)		15 (15.6)		3 (3.1)							
Symptomatic carotid		0.326		1.000		0.512						
Yes (*n = *18)	9 (50.0)		3 (16.7)		1 (5.6)							
No (*n = *93)	58 (62.4)		15 (16.1)		3 (3.2)							
Intracranial stenosis		0.400		1.000		1.000						
Yes (*n = *6)	5 (83.3)		1 (16.7)		0							
No (*n = *105)	62 (59.0)		17 (16.2)		4 (3.8)							
Atrial flutter		0.648		0.512		1.000						
Yes (*n = *4)	2 (50.0)		1 (25.0)		0							
No (*n = *107)	65 (60.7)		17 (15.9)		4 (3.7)							
Atrial fibrillation		0.004		0.012		1.000						
Yes (*n = *29)	11 (37.9)		9 (31.0)		1 (3.4)							
No (*n = *82)	56 (68.3)		9 (11.0)		3 (3.7)							
Aortic arch		1.000		0.299		1.000						
Yes (*n = *2)	1 (50.0)		1 (50.0)		0							
No (*n = *109)	66 (60.6)		17 (15.6)		4 (3.7)							
Cardiac heart failure		0.155		0.299		1.000						
Yes (*n = *2)	0		1 (50.0)		0							
No (*n = *109)	67 (61.5)		17 (15.6)		4 (3.7)							
PFO		0.155		1.000		1.000						
Yes (*n = *2)	0		0		0							
No (*n = *109)	67 (61.5)		18 (16.5)		4 (3.7)							
Extracardiac shunt		0.396		0.162		1.000						
Yes (*n = *1)	0		1 (100.0)		0							
No (*n = *110)	67 (61.5)		17 (15.5)		4 (3.6)							
Thrombolysis		0.751		0.021		1.000						
Yes (*n = *26)	21 (70.0)		8 (30.8)		1 (3.8)							
No (*n = *85)	46 (56.8)		10 (11.8)		3 (3.5)							
MES									0.547			16.83 (2.01–141)
Number of MES												
0	62 (60%)	0.429	16 (15.5%)	0,154	2 (1.9%)	0.017						
1	3 (100%)		0 (0%)		1 (33%)							
3	1 (50%)		0 (0%)		1 (50%)							
5	1 (33%)		2 (66%)		0 (0%)							

*NIHSS* National Institutes of Health Stroke Scale,* AMI* acute myocardial infarction,* CAD* coronary artery disease,* CHF* cardiac heart failure,* PFO* patent foramen ovale,* NC* not confounder for stroke recurrence

The absence of a statistically significant association was found between favorable outcomes (mRS 0–2) (*P* = 0.429), and death (*P* = 0.154) and positive association between the number of MES for patient and recurrence (*P* = 0.017). However, multivariate analysis was not performed due to the low number of patients with MES (great asymmetry of the sample). The OR for the outcomes as a function of the number of MES was analyzed for the total sample, and the analysis showed no statistical differences in terms of favorable outcomes for lower number of MES for patient (OR = 0.85 95% CI 0.56 to 1.30). The mortality (OR = 1.41 95% CI 0.92 to 2.16) and recurrence (OR = 1.55 95% CI 0.88 to 2.76) increased with the more number of MES for patient, but none of the three associations was statistically significant.

Four patients (3.6%) had a recurrence of stroke within 6 months of follow-up, and the TOAST classifications of these strokes were small vessels, cardioembolic, atherosclerosis of large vessels, and indeterminate nature, respectively. There were no confounding factors for stroke recurrence, and there was a significant relationship between the detection of MES and stroke recurrence during follow-up (OR = 16.83, 95% CI 2.01 to 141.08; *P* = 0.009).

**Fig. 3:**
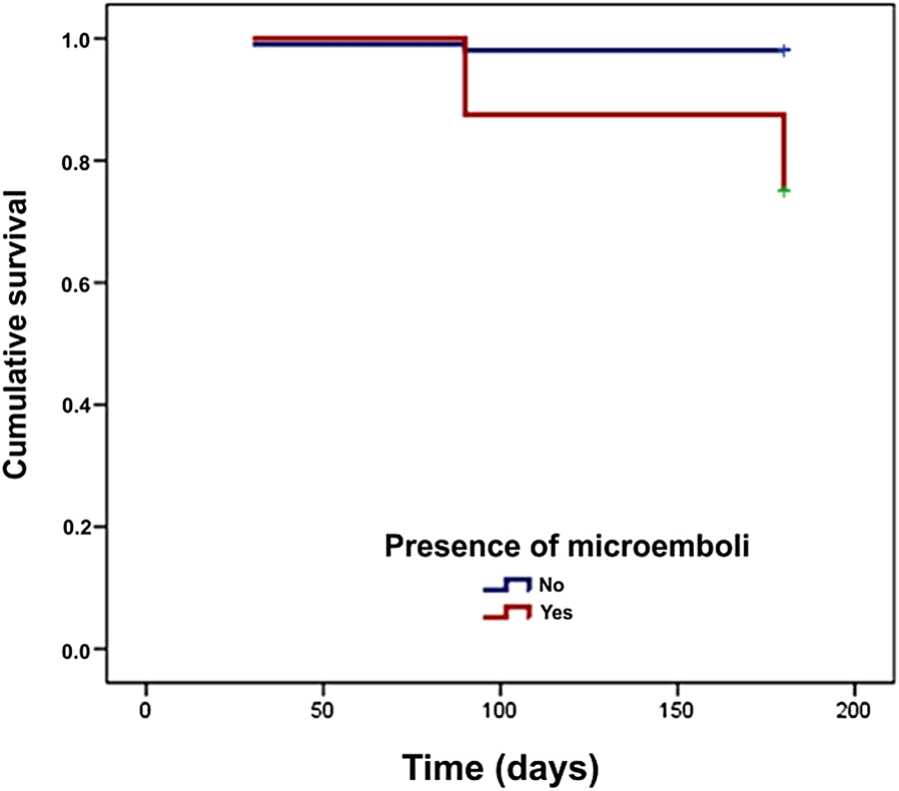
Survival curves according to the presence of MES corrected by prior stroke

Table 4 and Fig. 3 demonstrate the identified factors that were more strongly associated with the incidence rate of stroke recurrence.

**Table 4:** Simple regression models of Cox adjusted to the incidence rate of stroke recurrence due to each independent variable

Variable	*Β* estimate	Standard error	Wald test	*P*	Relative risk	CI95%	
Sociodemographic profile							
Sex, Male	0.57	1.16	0.25	0.620	1.77	0.18	17.0
Age (years)	0.00	0.04	0.00	0.988	1.00	0.93	1.1
Race, Non-white	− 3.33	5.48	0.37	0.543	0.04	0.00	1642.1
Risk factors							
Hypertension	3.28	5.85	0.31	0.576	26.47	0.00	3.E + 06
Smoking	0.95	1.16	0.67	0.412	2.58	0.27	24.8
Obesity	− 3.09	9.75	0.10	0.752	0.05	0.00	9.E + 06
Diabetes	0.99	1.00	0.98	0.323	2.69	0.38	19.1
Dyslipidemia	1.72	1.00	2.94	0.086	5.55	0.78	39.4
Chagas disease	− 3.12	8.49	0.14	0.714	0.04	0.00	8.E + 05
Prior stroke	1.87	1.16	2.62	0.106	6.48	0.67	62.3
Prior CHF	1.48	1.16	1.64	0.200	4.39	0.46	42.2
Prior AMI	1.12	1.16	0.94	0.331	3.07	0.32	29.5
Prior CAD	2.20	1.00	4.82	0.028	9.00	1.27	63.9
Clinical profile							
NIHSS	− 0.11	0.10	1.10	0.294	0.90	0.74	1.1
Previous mRS	0.18	0.52	0.12	0.729	1.20	0.44	3.3
Wake-up stroke	0.53	1.16	0.21	0.646	1.70	0.18	16.3
Time to monitoring < 24 h	1.52	1.16	1.73	0.188	4.57	0.48	43.9
Etiologic investigation							
Atrial fibrillation	− 0.06	1.16	0.00	0.957	0.94	0.10	9.0
Atrial flutter	− 3.06	11.87	0.07	0.797	0.05	0.00	6.E+08
CHF	− 3.03	16.69	0.03	0.856	0.05	0.00	8.E+12
PFO	− 3.03	16.69	0.03	0.856	0.05	0.00	8.E+12
Extracardiac shunt	− 3.01	23.54	0.02	0.898	0.05	0.00	5.E+18
Debris in aortic arch	− 3.03	16.69	0.03	0.856	0.05	0.00	8.E+12
Carotid atheromatous	0.74	1.16	0.42	0.519	2.11	0.22	20.2
Carotid dissection	− 3.03	16.69	0.03	0.856	0.05	0.00	8.E+12
Symptomatic carotid	0.60	1.16	0.27	0.605	1.82	0.19	17.5
Intracranial stenosis	− 3.09	9.75	0.10	0.752	0.05	0.00	9.E+06
Treatment							
Thrombolysis	0.08	1.16	0.01	0.942	1.09	0.11	10.5
MES							
Presence of MES	2.58	1.00	6.65	0.010	13.18	1.86	93.64

CI: confidence interval; CHF: cardiac heart failures; AMI: acute myocardial infarction; CAD: coronary artery disease; NIHSS: National Institutes of Health Stroke Scale; mRS: modified Rankin scale; PFO: patent foramen ovale

It was observed that the stroke recurrence adjusted for prior stroke was higher and earlier among patients with MES detection (RRadjusted = 36 (4–323), *P* < 0.001).

During hospitalization and after discharge, 16 patients started using anticoagulant therapy, while all others were given antiplatelet therapies. The treatment of carotid artery disease was performed in 5 patients exclusively with endovascular therapy (stent). No patient was treated with endarterectomy due to an intrinsic characteristic of the hospital.

## Discussion

Of the total number of patients involved in this study, 26% did not have a TAW on TCD examination. These results were demonstrated in a previous study with the same sample by our stroke team [22]. The other studies in the literature showed a range of 3–34% absence of acoustic window, including populations from healthy volunteers and patients with cerebrovascular disease [23].

The MES detected by TCD have been described in patients with carotid artery stenosis, dissection of large cervical vessels, atrial fibrillation, catheter-based procedures, and atheromatous plaques in the aortic arch, but their clinical application remains uncertain [24, 25]. The number of patients with MES in this study was 8 patients (7%). Other samples showed variations in the rate of MES detection in the acute phase of stroke ranging between 9.3 and 71%, with most studies demonstrating a relationship with large artery disease and less frequently with cardioembolic disease [26–29]. MES were not detected in cases with intracranial stenosis, in contrast to a prevalence of 22% reported in other studies [3]. The high percentage of patients who underwent thrombolytic treatment or who were using antiplatelet therapies during transcranial Doppler monitoring, despite not presenting a statistically significant correlation with the detection of MES, may have influenced the low detection rate of these MES. However, it must be emphasized that these monitoring conditions in the acute and subacute stages of a stroke correspond to the conditions that most resemble the real world.

A statistically significant relationship between the MES and patients with large vessel disease was found. There was also a significant association between detecting MES and symptomatic carotid artery disease. Of patients with symptomatic carotid disease, two had arterial dissection at the time of monitoring by TCD, and in one of these, MES were found. Some other studies have demonstrated the importance of microembolism in TCD monitoring as a risk factor for stroke in carotid dissection [30–32]. One of the patients examined in the present study was diagnosed with pulmonary thromboembolism and left-to-right shunts could be detected with a simple bubble test, without contrast. This patient had extensive ischemia in the territory of both the middle cerebral arteries with bilateral MES during TCD monitoring, a relatively rare finding in the literature [33]. TCD associated with the use of contrast is a useful tool in the investigation of right-left deviations, such as patent foramen ovale and other rare medical conditions, such as pulmonary fistula.

This study showed a higher chance of MES detection the sooner the monitoring time was in relation to the onset of symptoms of stroke. Studies conducted in the 1990s showed that early monitoring with TCD in acute stroke increases the likelihood of the detection of MES [34].

No relationship was found between the detection of MES and the degree of disability and death; correlations were found with aging, NIHSS, and prior mRS. These findings were similar to other studies in the literature [6, 34]. In this study, smoking seemed to be a protective factor. This paradoxical finding was shown in other studies and can be explained by the better vascular recanalization in patients who smoke [35].

The presence of MES showed a good correlation with early stroke recurrence. A study that analyzed 467 patients showed that the risk of ischemic stroke occurrence increased 5.57 times over the course of 2 years of follow-up when MES were identified and that when MES were detected, there was a greater chance of new cerebral ischemic events occurring [27, 28]. In a meta-analysis from 2009, the authors found a twofold increase in the chance of new cerebral ischemic events when they identified embolic signals in TCD monitoring [3].

In relation to the recurrence of cerebral ischemic events, the present study showed a relationship between coronary artery disease (CAD) and stroke recurrence, demonstrating the probable role of systemic atherosclerotic disease and the role and coexistence of atherosclerotic cardiac comorbidities in these patients, even though we cannot rule out the presence of hidden atrial fibrillation in patients with high burden of atherosclerotic diseases. The low rate of stroke recurrence at 6 months of follow-up may be justified by the clinical and endovascular therapies instituted.

### Clinical implications

Early detection of MES in acute phase of stroke may be a clinical indicator to determine carotid disease and recurrences of stroke. This indicator should promptly request a confirmation of possible carotid disease with other methods.

## Limitations and strengths

The main limitation of this study is the small number of patients included, as well as the non-inclusion of patients with strokes in the posterior circulation. Also, a limitation of TCD is that the TAW can be obtained from a variable percentage of patients, as well as the fact that this technique is operator dependent. Other limitations of this study are related to the characteristics of the recruited patients (the study was performed in a single center), in addition to technical difficulties with the settings of the monitoring helmet, which generated a greater number of artifacts. The fact that the patients were in the acute phase of stroke and the presence of neurological symptoms, such as unilateral spatial neglect, aphasia, psychomotor agitation, or delirium, made TCD monitoring difficult. In relation to the MES detection points, care must be taken during the examination and the interpretation of the findings due to the need to discriminate MES from possible artifacts.

## Conclusion

In conclusion, it was found that MES detection showed a higher correlation with symptomatic carotid disease and a shorter time between ictus and TCD monitoring. In addition, the presence of MES and CAD could be predictors for stroke recurrence.

## Data Availability

Data are available with the main author, who can provide all information in case of the editor’s requirement.
